# Thermodynamic insight into the growth of calcia inclusions at the nanoscale: the case of Fe–O–Ca melt†

**DOI:** 10.1039/c9ra01337g

**Published:** 2019-04-09

**Authors:** Yuanyou Xiao, Hong Lei, Bin Yang, Yang Zhao, Qi Wang, Guocheng Wang

**Affiliations:** Key Laboratory of Electromagnetic Processing of Materials, Ministry of Education, Northeastern University Shenyang Liaoning Province 110819 P. R. China wang_guocheng@163.com; School of Metallurgy, Northeastern University Shenyang Liaoning Province 110819 P. R. China; School of Materials and Metallurgy, University of Science and Technology Liaoning Anshan Liaoning Province 114051 P. R China

## Abstract

A thermodynamic model was developed to investigate the relationship between the thermodynamics of nano-CaO as a deoxidation reaction product and their size in an Fe–O–Ca melt. The results of thermodynamic model coupling with DFT (density functional theory) calculation prediction showed that the solubility product of calcium and oxygen for nanoscale CaO decreased with the increase of calcia product size in an Fe–O–Ca melt. The existing experimental data about the Ca-deoxidation equilibrium in liquid iron are covered by the region between the bulk-calcia equilibrium curve and the nano-CaO of 2 nm size curve. This result indicates that the partial product in most of the Ca-deoxidation experiments could be nanoscale CaO particles. Most of the Ca-deoxidation experimental equilibrium states are not reaching the equilibrium state between bulk calcia and liquid iron but a multi-equilibria between bulk- and nano-CaO and liquid iron.

## Abstract 2

Thermodynamics of the formation of nano-CaO in liquid iron is important to explore the relationship between deoxidation reaction and size of inclusions, and is very useful in the size controlling of inclusions.

## Graphical abstract

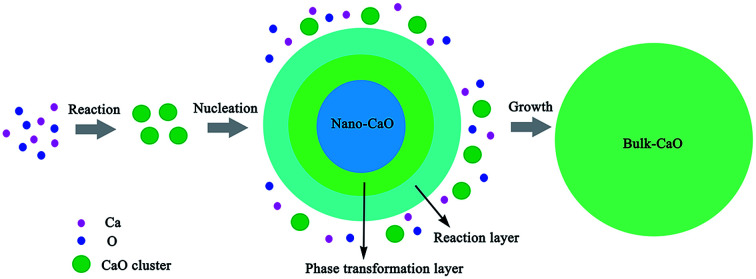

## 1. Introduction

Non-metallic inclusions are an important factor that affects the quality of steel products because their properties differ from those of the steel matrix, and they act as stress raisers and a source of cracks. It is very difficult to eliminate all inclusions during the steelmaking process. In order to reduce the harm of inclusions, metallurgists make their best efforts to transform the sharp inclusions with high melting point to small curved inclusions by magnesium treatment^1–5^ or liquid (or partially liquid) calcium treatment.^5–12^ On the other hand, inclusion size control is one of the effective measures to improve steel performance.^1^ Oxide inclusions, which come from the products of metal deoxidation in molten steel, are very common in steel. In order to control the size of oxide inclusions, it is necessary to have a deep insight into the thermodynamics of inclusion during deoxidization of molten steel.

Calcium is a popular deoxidizer during the steelmaking process because of the strong affinity with oxygen, and its thermodynamic property is important in estimating an optimum operation condition. The reaction equation of Ca-deoxidation for molten steel to generate bulk calcia by dissolved Ca and O can be written as

1 [Ca] + [O] = CaO_(bulk)_

The equilibrium constant of Ca-deoxidation reaction can be expressed as^13^2
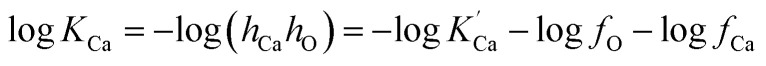
where 
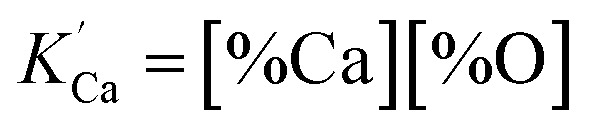
 is the solubility product; *h*_i_ and *f*_i_ are the activity of element i in the molten iron and its activity coefficient relative to an infinitely dilute solution on a mass percentage basis, respectively. The *f*_i_ can be expressed as^13^

3 log *f*_Ca_ = *e*^Ca^_Ca_[% Ca] + *e*^O^_Ca_[% O] + *r*^Ca^_Ca_[% Ca]^2^ + *r*^O^_Ca_[% O]^2^ + *r*^O,Ca^_Ca_[% O][% Ca]

4 log *f*_O_ = *e*^O^_O_[% O] + *e*^Ca^_O_[% Ca] + *r*^O^_O_[% O]^2^ + *r*^Ca^_O_[% Ca]^2^ + *r*^Ca,O^_O_[% O][% Ca]

where *e*^i^_i_ and *e*^j^_i_ are the first-order interaction coefficients; *r*^i^_i_, *r*^j^_i_ and *r*^i,j^_i_ are the second-order interaction coefficients.

The Ca-deoxidation equilibrium thermodynamics in liquid iron at 1873 K has been investigated for many years. Unfortunately, there is no exact thermodynamic parameters for calcium deoxidation reactions in molten steel because the calcium is easily vaporized at high temperature process. Table 1 lists the measured equilibria constants and the interaction coefficients between calcium and oxygen in liquid iron at 1873 K.^14–21^ Ototani *et al.*,^16^ Gustafsson *et al.*,^17^ and Kimura *et al.*^18^ estimated the values of equilibria constants and the first-order interaction coefficients at l873 K. Considering of the strong interaction between Ca and O in liquid iron, Wakasugi *et al.*,^19^ Cho and Suito,^20^ and Itoh *et al.*,^21^ obtained their equilibria constants with a second-order interaction coefficients based on the experimental data. Specifically, in order to express the calcium-oxygen equilibrium, Kimura *et al.*^18^ estimated the values of *K*_Ca_ and the interaction coefficients depending on the values for {[% Ca] + 2.51[% O]} in three different ranges by using the first-order interaction coefficients only. By fixing the value of log *K*_Ca_, Cho and Suito^20^ used the multiple regression analysis to determine the values for the first-order interaction coefficients and the second-order interaction coefficients, which obtained as −3600 and 5.7 × 10^5^ in the range of {[% Ca] + 2.51[% O]} < 0.005 and −990 and 4.2 × 10^4^ in the range of {[% Ca] + 2.51[% O]} > 0.005, respectively. However, the equilibrium constant and the interaction coefficients proposed by different researchers are different from each other. As listed in Table 1, the minimum value of the first interaction coefficients *e*^Ca^_O_ (−5000) is far less than the maximum (−60), and the minimum value of the second interaction coefficients *r*^Ca^_O_ (−18 000) is far less than maximum (570 000). The minimum and the maximum of log *K*_Ca_ by thermodynamic calculations are 6.05 and 10.04,^14,15^ and the minimum and the maximum of log *K*_Ca_ by experiments are 5.8 and 10.3.^17,18^ It should be note that the equilibrium constants and the interaction coefficients obtained by various researchers are different from each other and even vary widely. Consequently, it is not easy to select the suitable equilibrium constant and the interaction coefficients. Such a strange phenomenon puzzled the researchers for years.

**Table 1:** Equilibrium constants and interaction coefficients of Ca–O system in liquid iron at 1873 K

	log *K*_Ca_	*e* ^Ca^_O_	*e* ^O^_Ca_	*r* ^Ca^_O_	*r* ^O^_Ca_	*r* ^Ca,O^_O_	*r* ^Ca,O^_Ca_
**Thermodynamic calculation**							
Nadif *et al.*^14^	6.05	—	—	—	—	—	—
Kulikov^15^	10.04	—	—	—	—	—	—
**Experiment result**							
Ototani *et al.*^16^	8.23	−535	−1330	—	—	—	—
Gustafsson *et al.*^17^	5.8	−62	−155	—	—	—	—
Kimura *et al.*^18^^,^^c^	10.3	−5000	−12 550	—	—	—	—
Kimura *et al.*^18^^,^^d^	7.6	−600	−1506	—	—	—	—
Kimura *et al.*^18^^,^^e^	5.8	−60	−150	—	—	—	—
Wakasugi *et al.*^19^	9.4	−1400	−3500	8500	53 000	43 000	43 000
Cho *et al.*^20^^,^^a^	10.22	−3600	−9000	570 000	3 600 000	2 900 000	210 000
Cho *et al.*^20^^,^^b^	10.22	−990	−2500	42 000	260 000	2 900 000	210 000
Itoh *et al.*^21^	7.15	−310	−780	−18 000	650 000	520 000	−90 000

a [% Ca] + 2.51[% O] < 0.005.

b [% Ca] + 2.51[% O] = 0.005 to 0.018.

c [% Ca] + 2.51[% O] < 0.0008.

d [% Ca] + 2.51[% O] = 0.0008 to 0.003.

e [% Ca] + 2.51[% O] > 0.003.

It was reported that there is a close relationship between the thermodynamic properties and the size of deoxidation product particles.^22,23^ The products of metal deoxidation reaction in liquid iron in most case could be stable and metastable inclusions.^22,23^ Therefore, the difference of thermodynamics for Ca-deoxidation in liquid iron may be caused by the size effect of inclusion product. Nano-calcia is the intermediate product of the crystallization for bulk calcia inclusion during Ca-deoxidation process. Understanding the thermodynamics of nano-calcia is important to explore the relationship between the size of calcia inclusions and Ca-deoxidation reaction in liquid iron. In this paper, a thermodynamic model was developed to investigate the relationship between the thermodynamics of nano-calcia and their size in a Fe–O–Ca melt.

## 2. Theoretical modeling for nano-CaO in liquid iron

### 2.1. Thermodynamic modeling

The nano-particle can be considered as two parts:^24–31^ an internal part (atoms located in the lattice of crystallites) and an external part (atoms situated in the particle surface). During the calculation of the thermodynamic properties of nano-CaO particle, the contributions of both parts should be considered separately. The thermodynamic properties of nano-CaO can be obtained *via A*_n_ = (1 − *x*_s_)*A*_i_ + *x*_s_*A*_s_,^24,25^ where *A*_s_ is the thermodynamic property of the external part of nano-CaO, *A*_i_ is the thermodynamic property of the internal part of nano-CaO, *x*_s_ is the atomic fraction in the surface of nano-CaO.

**Fig. 1:**
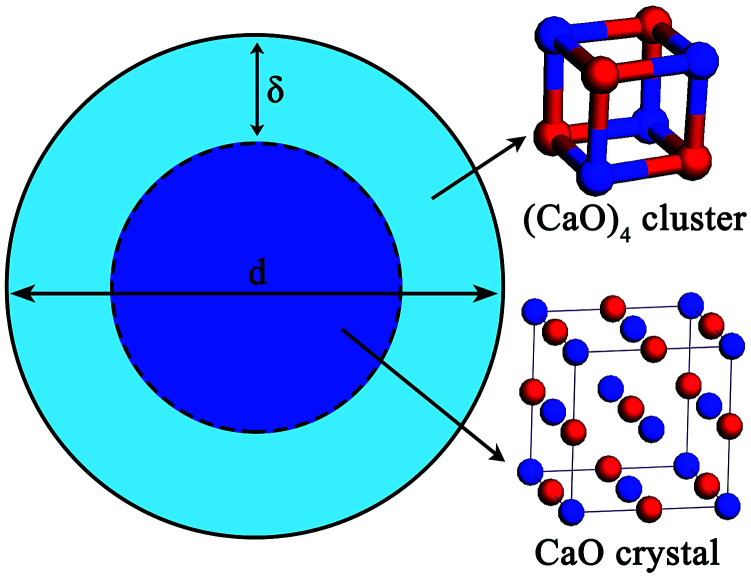
Structure model of nano-CaO.

In this work, the nano-CaO, which is a sphere particle with diameter *d*, contains a surface of *δ* thickness and a core with diameter (*d* − 2*δ*), as schematically shown in Fig. 1. The atomic fraction in the surface of nano-CaO can be expressed as^24^5
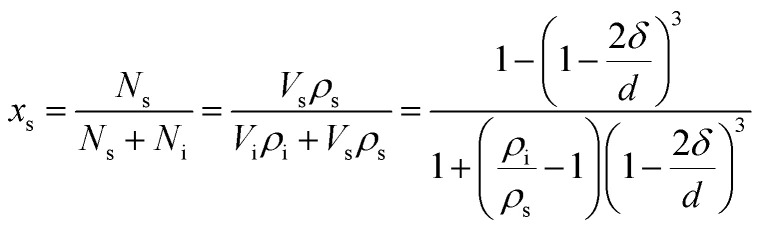
where *N*_i_ and *N*_s_ are the atom numbers at the inner and surface of nano-CaO, respectively; *ρ*_i_ is the atomic densities of internal part of nano-CaO; *ρ*_s_ is the atomic densities of surface part of nano-CaO.

**Fig. 2:**
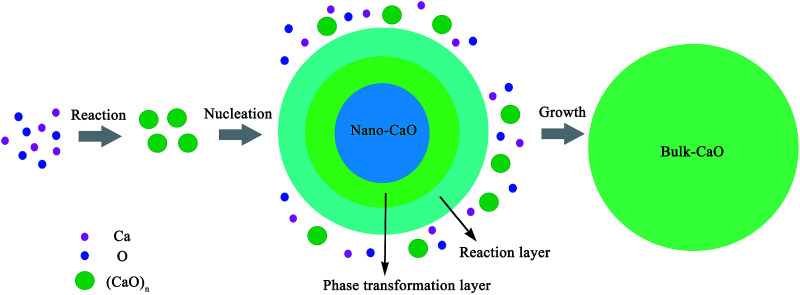
Schematic of the nucleation and growth of CaO inclusion.

Experimental studies indicated that the atomic density at a particle surface is lower than that of the perfect crystal by 10–30%.^26,27^ Thus, the value of *ρ*_i_/*ρ*_s_ was taken as 1.2 in the current calculation. Nano-CaO is the intermediates of CaO product particle growth in the Ca-deoxidation reaction, and its surface is formed by the aggregation and phase transformation of (CaO)_*n*_ clusters as shown in Fig. 2. Some studies^25,28^ about surface structures reported that there is a liquid-like layer but not a real liquid layer on a variety of surfaces below the melting point, and the crystalline order will gradually decrease in the liquid-like layer from the bulk side to the free surface. Thomas *et al.*^29^ proved that the nano-crystalline interface is short-range order structure by high-resolution electron transmission microscopy. Therefore, the surface structure of the nano-CaO is a short-range order structure and is similar to the structure of (CaO)_*n*_ clusters. In order to simplify our modeling, we can use the (CaO)_*n*_ cluster to describe the particle surface structure. Moreover, Waniewska *et al.*^30^ reported that the surface of nano-particle usually contains 2 or 3 atom layers. In this work, the surface of nano-CaO was taken as 2 atom layers. Hence, the calcia cluster (CaO)_4_, which contains two-atom thick, can be used to describe the particle surface structure, and the calcia crystal CaO_(bulk)_ can be used to describe the structure of particle internal part as shown in Fig. 1. As a result, the thermodynamic properties of the internal part of nano-CaO and the external part of nano-CaO are the same as that of (1/4) (CaO)_4_ and CaO_(bulk)_, respectively. Phillpot *et al.*^31^ suggested that the thickness of grain boundary is generally about 2.5–3.5 times of their lattice parameter. Therefore, the thickness of the nano-CaO particle surficial layer *δ* was taken as 0.5 nm in current calculation. The atomic fractions of the surface components are shown in Table 2.

**Table 2:** Atomic fractions of the surface components of nano-CaO

*d* (nm)	2	3	4	5	6	7	8	9
*x*_s_ (%)	85.37	66.43	53.31	44.27	37.76	32.88	29.11	26.10
*d* (nm)	10	15	20	30	40	50	100	200
*x*_s_ (%)	23.65	16.08	12.17	8.19	6.17	4.95	2.49	1.25

### 2.2. DFT calculation details

The thermodynamic properties of (CaO)_4_ cluster and CaO_(bulk)_ crystal were calculated by Dmol3 module of Materials Studio 7.0, a molecular orbital theory computational program, which was based on density functional theory. The geometry optimization for (CaO)_4_ cluster and CaO_(bulk)_ crystal were performed using the BFGS (the abbreviation corresponds to the first letters of the names of the following researchers: Broyden, Fletcher, Goldfarb, and Shanno) method^32^ based on a quasi-Newton algorithm. The framework of the generalized gradient approximation (GGA) proposed by Perdew, Burke, and Ernzerhof^33^ was used as the exchange–correlation potential function during the calculations. The values of the convergence thresholds for the maximum energy change, the maximum force and the maximum displacement are given as 1 × 10^−5^ Ha, 0.002 Ha Å^−1^ and 0.005 Å, respectively. The self-consistent field (SCF) method was used to control the electronic minimization and the threshold for the total energy and SCF density convergence set as 1 × 10^−6^ Ha. In order to reduce the computational cost, electrons outside the atomic nucleus were handled by the all-electron method. The atomic orbital basis set was double numeric quality with polarization functions (DNP). The cut-off radius of the DNP basis set of the d orbital was 3.5 Å. The thermodynamic properties of (CaO)_4_ cluster and CaO_(bulk)_ crystal were calculated by using the atomic harmonic vibrational frequency. The enthalpy (*H*) and entropy (*S*) were obtained by the vibrational analysis as functions of temperature. The details of the relationship among the thermodynamic properties, the atomic harmonic vibrational frequency and temperature can be found in the previous study.^23^

## 3. Results and discussion

### 3.1. Thermodynamic properties of nano-CaO

The calculated thermodynamic properties of (CaO)_4_ cluster, CaO_(bulk)_ and nano-CaO from 1000 K to 2000 K are shown in Tables S1–S3.† Both the *H* and *S* of (1/4) (CaO)_4_ cluster, CaO_(bulk)_ and nano-CaO increase with the increasing of temperature. The *H* of (1/4) (CaO)_4_ cluster, CaO_(bulk)_ and nano-CaO increase with the increasing of calcia size, while their *S* decrease with the increasing of calcia size. The Gibbs free energy can be calculated by the equation, *G* = *E* (0 K) + *H* − *TS*, where *E* (0 K) is the total energy at 0 K. The numerical results of *E* (0 K) for (CaO)_4_ and CaO_(bulk)_ are −1 976 455.155 kJ mol^−1^ and −1 976 116.107 kJ mol^−1^, respectively. While the Gibbs free energy of (1/4) (CaO)_4_ cluster, CaO_(bulk)_ and nano-CaO decreases with the increasing of temperature and increasing of calcia size. This result indicates that the thermodynamic stabilities of CaO particles with different sizes increase with the increasing of temperature, the stabilities of CaO particles increase with the increasing of size, and the CaO_(bulk)_ is more stable than nano-CaO at the same temperature.

### 3.2. Gibbs free energy changes for the formation of nano-CaO in liquid iron

The formation of nano-CaO in liquid iron can be described as

6 [Ca] + [O] = nano-CaO

The Gibbs free energy change for this step is Δ*G*_*n*_(6). Then, nano-CaO continues to grow into stable bulk CaO inclusions as

7 nano-CaO → CaO_(bulk)_

**Fig. 3:**
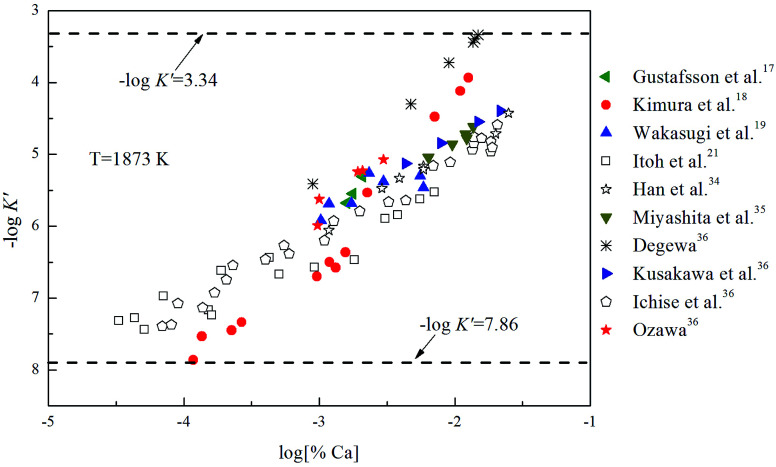
Solubility product of calcium and oxygen for nano-CaO in liquid iron at 1873 K.

The Gibbs free energy change for this step is calculated Δ*G*_*n*_(7) = *G*_CaO_(bulk)__ − *G*_*n*_, where *G*_CaO_(bulk)__ is Gibbs free energy of CaO_(bulk)_, *G*_*n*_ is Gibbs free energy of nano-CaO with size of *n* nm. Therefore, 
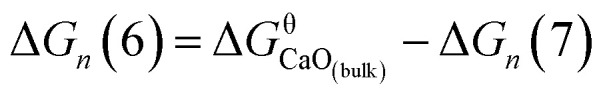
, where 
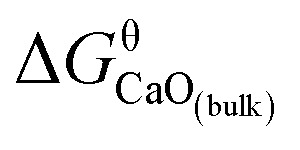
 is Gibbs free energy change of eqn (1). The Fe–O–Ca melt is similar to the infinite dilute solution because of the concentrations of [O] and [Ca] at a very low range. Thus, the value of log *K* is close to −log *K*′. As shown in Fig. 3, the solubility product of calcium and oxygen for nano-CaO in liquid iron from the experimental measurement at 1873 K are different from each other, and the value of −log *K*′ ranges from 3.34 to 7.86. In this work, the equilibrium constant of bulk calcia equilibrated in liquid iron at 1873 K is taken as log *K* = 7.86. The value of 
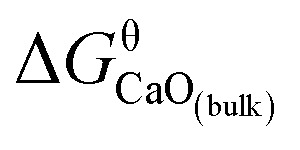
 can be estimated as 
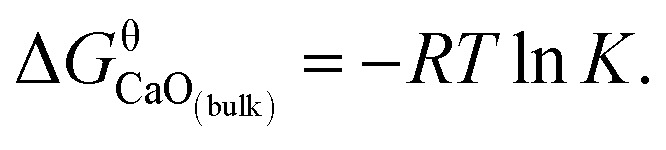


As shown in Table 3, the value of Δ*G*_*n*_(6) decreases with the increasing size of nano-CaO product. The solubility product of calcium and oxygen for nano-CaO in liquid iron called as 
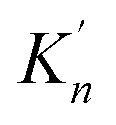
, and the value of 
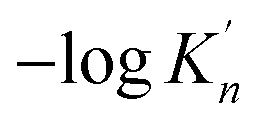
 ranges from 2.43 to 7.86 is similar to the value of −log *K*′. Moreover, the value of 
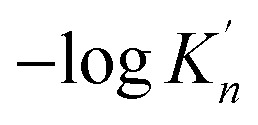
 increases with the increasing size of nano-CaO product. Such a result indicates that the thermodynamic relationship between the nano-CaO and liquid iron are gradually close to the final equilibrium between bulk calcia and liquid iron with the increasing calcia products size during the growth process. This is the reason that the equilibrium constants obtained by various researchers are different from each other.

**Table 3:** Gibbs free energy changes for nano-CaO forming in liquid iron at 1873 K

*d* (nm)	2	3	4	5	6	7	8	9
Δ*G*_*n*_(6) (kJ mol^−1^)	−87.141	−130.334	−160.254	−180.870	−195.717	−206.845	−215.443	−222.307
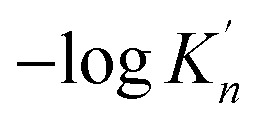	2.43	3.63	4.47	5.04	5.46	5.77	6.01	6.2
*d* (nm)	10	15	20	30	40	50	100	200
Δ*G*_*n*_(6) (kJ mol^−1^)	−227.895	−245.158	−254.075	−263.152	−267.758	−270.540	−276.151	−278.978
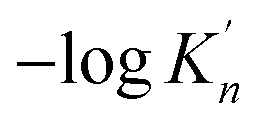	6.36	6.84	7.09	7.34	7.47	7.55	7.7	7.78

### 3.3. Multi-equilibria thermodynamics of CaO in liquid iron

**Fig. 4:**
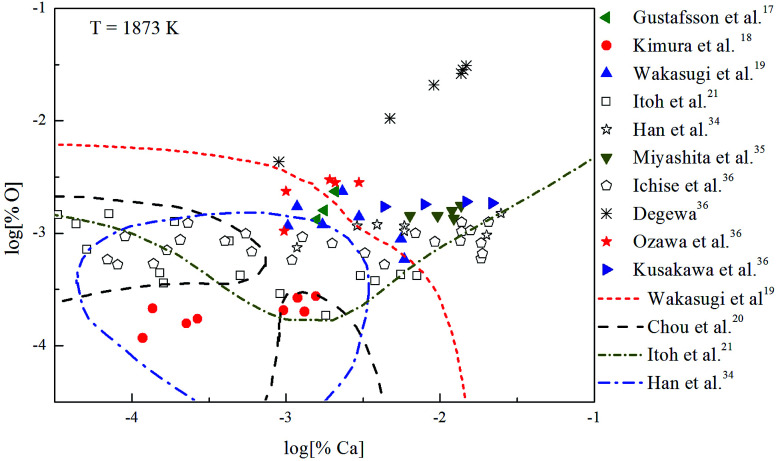
Equilibrium experiments of Ca-deoxidation in liquid iron at 1873 K.

The Ca-deoxidation equilibrium in liquid iron have been intensely studied by many researchers.^17–21,34–36^ By using a high argon pressures to reduce calcium losses, Gustafsson *et al.*^17^ performed their experiments in an open containers. In order to help the system to be much closer equilibrium state, Itoh *et al.*^21^ carried out their experiments in a high frequency induction furnace with open dolomite crucibles under a mixture atmosphere of argon and hydrogen. Kimura *et al.*^18^ performed Ca-deoxidaton equilibrium experiments in a vertical resistance furnace with heating bars of LaCrO_3_ in Al_2_O_3_ and CaO crucibles. The experimental measured data of Ca-deoxidation equilibrium in liquid iron at 1873 K are shown in Fig. 4. The equilibrium concentration of [Ca] is fluctuating within the range of 3.2 × 10^−5^ < [% Ca] < 0.032 in equilibrium with solid CaO. Meanwhile, the equilibrium concentration of [O] decreases with the increasing [Ca] when the equilibrium concentration of [% Ca] < 0.001, but the equilibrium concentration of [O] increases with the increasing [Ca] in the case of the equilibrium concentration of [% Ca] > 0.001. It should noted that the difference among the equilibrium concentrations of [O] proposed by different researcher at the same concentration of [Ca] is more than one order of magnitude.

Many researchers^19–21,34^ gave their thermodynamic equilibrium curves to describe the thermodynamic equilibrium of Ca-deoxidation for liquid iron at 1873 K. As shown in Fig. 4, the Ca-deoxidation equilibrium relation proposed by Cho *et al.*^20^ shows a Ω-shaped curve, while the equilibrium relation obtained by Han *et al.*^34^ shows a oval-shaped curve. This means one concentration of calcium corresponds to two or more concentrations of oxygen and *vice versa*. The equilibrium relation of Ca-deoxidation obtained by Wakasugi *et al.*^19^ had a decreasing trend even in the case of the equilibrium concentration of [% Ca] > 0.001 in liquid iron. In addition, the equilibrium relation of [Ca] and [O] obtained by Itoh *et al.*,^21^ have a “V” shape. Their equilibrium curves descend with the increasing of Ca concentration and agree well with their equilibrium experiment data in the case of the equilibrium concentration of [% Ca] < 0.001 in liquid iron. It is thus clear that, there is not a same curve to describe these equilibria experimental results data of Ca-deoxidation for liquid iron.

Previous report showed^37^ that the [Ca] and [O] could not be independent randomly distributed, but had a strong tendency to form dissolved associated compound Ca–O *etc.* as a kind of metastable phase. Hence, it can be suggested that the thermodynamic equilibrium of Ca-deoxidaiton reaction in liquid iron has a close relationship with the metastable phase, such as dissolved associated compound Ca–O, nano-CaO *etc.* Wang *et al.*^22,38,39^ reported that the deoxidation thermodynamics in liquid iron have a close relationship with the structures and properties of deoxidaiton products, and suggested that the deoxidizers react with dissolved oxygen in molten steel to form various metastable intermediates at first, and then the metastable intermediates transform into stable crystal inclusion. Wasai *et al.*^40^ and Zhao *et al.*^41^ found nanoscale inclusions in deoxidation experiments by ultra-rapid cooling process. Therefore, the metastable calcia form at first in the process of Ca-deoxidation as shown in Fig. 2. According to the Ostwald's step rule,^42^ the nano-CaO can grow into bulk calcia by attracting the surrounding [Ca], [O] and small calcia (such as (CaO)_*n*_ clusters) in reaction layer. However, the deoxidation reaction in liquid iron is very difficult to reach the final equilibrium between bulk inclusion and liquid iron because of the decreasing of supersaturation of O and Ca. The supersaturation ratio *S* for the formation of solid calcia can be written as *S* = {[% Ca][% O]}/{[% Ca]_eq_[% O]_eq_}, where [% Ca] and [% O] are the experimental values; [% Ca]_eq_ and [% O]_eq_ are the equilibrium values. As the Ca-deoxidation reaction proceeds, the thermodynamic driving force decreased gradually with the decreasing of the supersaturation ratio in Ca-deoxidation process. Therefore, it is very difficult for the nano-CaO to grow into the final bulk calcia at the later deoxidation period. The collision probability is low and the nano-CaO are not large enough to float up. As a result, the nano-CaO appears as the structural units in Ca-deoxidation reaction in the liquid iron, and may remain as suspending inclusions for a long time. The products of Ca-deoxidation reaction may be various nano-CaO inclusions in many cases. Many studies^43–48^ have given the nano-scale particle's abnormal behavior with regard to thermodynamic property and structure stability, and these anomalies are caused by the remarkable difference between the interfacial structure and the bulk structure. Therefore, the thermodynamic difference of Ca-deoxidation proposed by different researchers may come from the different metastable calcia.

**Fig. 5:**
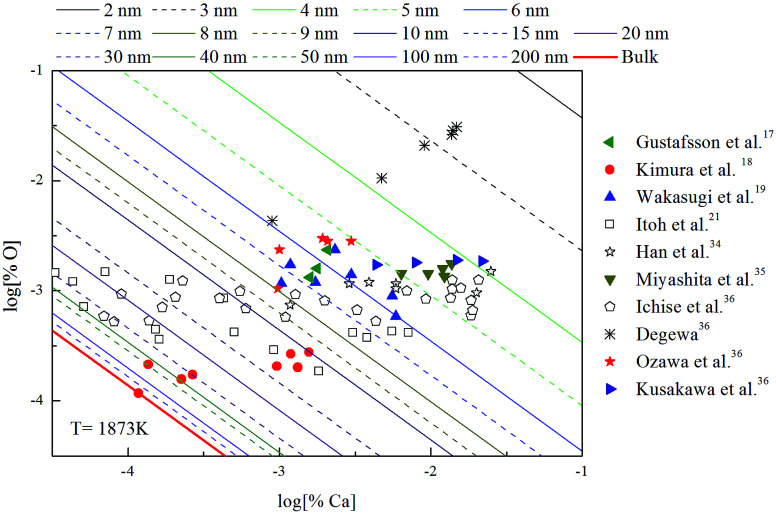
Equilibrium curves of nano-calcia equilibrated in liquid iron at 1873 K.

The thermodynamic curves of nano-CaO in liquid iron during the Ca-deoxidation process at 1873 K based on the above calculated results are shown in Fig. 5. All the experimental data are covered by the region between thermodynamic curves of 2 nm calcia and bulk-calcia. This result suggests that the experiments by various researchers are in different thermodynamic state. Some of these experiments are in equilibrium with bulk-calcia, while most of these experiments are in meta-stable equilibrium state (or quasi-equilibrium state). It suggested that the reaction product should be nanoscale calcia but not bulk-calcia in most of those experiments. The nano-CaO curves are close to the bulk-calcia equilibrium curve gradually with the increase of calcia inclusion size. This is the reason that the various previous Ca-deoxidation experimental data are different to each other even in case of the same concentration of [Ca].

## 4. Conclusions

The present work developed a thermodynamic modeling to investigate Ca-deoxidation reaction between nano-CaO inclusion and liquid iron. The following conclusions are obtained.

(1) The solubility product of calcium and oxygen for nano-CaO in liquid iron increased with the increasing calcia products size. The experimental data about Ca-deoxidation in liquid iron are covered by the region between the bulk-magnesia equilibrium curve and the nano-CaO curve of 2 nm.

(2) The previous Ca-deoxidation experiments are in the different thermodynamic states, and most previous experiments are in the quasi-equilibrium but not reach the final equilibrium because their partial product is nano-CaO. This is the reason that the various previous Ca-deoxidation experimental data are different to each other even in case of the same concentration of [Ca].

## Conflicts of interest

There are no conflicts to declare.

## Supplementary Material

RA-009-C9RA01337G-s001
