# Supplementary material for: Thermodynamic insight into the growth of calcia inclusions at the nanoscale: the case of Fe–O–Ca melt
Source: RSC Adv. 2019 Apr 9;9(20):11135–41. doi: 10.1039/c9ra01337g (PMC9063027; doi:10.1039/c9ra01337g)
Supplement: RA-009-C9RA01337G-s001 [file RA-009-C9RA01337G-s001.pdf]

Electronic Supplementary Material (ESI) for RSC Advances.
This journal is © The Royal Society of Chemistry 2019

Supplementary information

Table S1 The enthalpy (*H*) for (CaO)4, CaO(bulk) and nano-CaO

T	(1/4)$(CaO)_4$	2 nm	3 nm	4 nm	5 nm	6 nm	7 nm	8 nm	9 nm
1000	46.669	47.271	48.050	48.589	48.961	49.228	49.429	49.584	49.708
1100	51.158	51.817	52.672	53.263	53.671	53.964	54.184	54.354	54.490
1200	55.660	56.378	57.308	57.952	58.395	58.715	58.954	59.139	59.287
1300	60.173	60.950	61.955	62.651	63.131	63.477	63.736	63.936	64.096
1400	64.694	65.529	66.610	67.359	67.876	68.247	68.526	68.741	68.913
1500	69.223	70.117	71.274	72.075	72.628	73.025	73.323	73.554	73.738
1600	73.757	74.709	75.942	76.796	77.385	77.809	78.126	78.372	78.568
1700	78.295	79.306	80.615	81.522	82.147	82.597	82.934	83.195	83.403
1800	82.837	83.907	85.292	86.252	86.913	87.389	87.746	88.022	88.242
1873	86.473	87.590	89.036	90.038	90.728	91.225	91.598	91.886	92.116
1900	87.383	88.512	89.973	90.985	91.683	92.185	92.562	92.853	93.085
2000	91.931	93.119	94.656	95.721	96.455	96.984	97.380	97.686	97.930
	10nm	15 nm	20 nm	30 nm	40 nm	50 nm	100 nm	200 nm	CaO(bulk)
1000	49.809	50.120	50.281	50.444	50.527	50.578	50.679	50.730	50.781
1100	54.601	54.942	55.118	55.298	55.389	55.444	55.555	55.611	55.667
1200	59.407	59.779	59.971	60.166	60.265	60.325	60.446	60.507	60.568
1300	64.226	64.628	64.835	65.046	65.154	65.218	65.349	65.415	65.481
1400	69.053	69.485	69.708	69.935	70.051	70.120	70.261	70.331	70.403
1500	73.887	74.350	74.588	74.832	74.955	75.029	75.180	75.256	75.332

1600	78.727	79.220	79.475	79.734	79.865	79.945	80.105	80.186	80.267
1700	83.572	84.096	84.366	84.641	84.781	84.865	85.035	85.121	85.207
1800	88.421	88.975	89.261	89.552	89.700	89.789	89.969	90.060	90.151
1873	92.303	92.881	93.180	93.483	93.638	93.731	93.919	94.013	94.109
1900	93.274	93.858	94.160	94.467	94.623	94.717	94.906	95.002	95.099
2000	98.129	98.744	99.061	99.384	99.548	99.647	99.847	99.948	100.049

Table S2 The entropy (S) for (CaO)4, CaO(bulk) and nano-CaO

T	(1/4)$(CaO)_4$	2 nm	3 nm	4 nm	5 nm	6 nm	7 nm	8 nm	9 nm
1000	148.768	140.355	129.464	121.920	116.721	112.978	110.171	108.003	106.273
1100	156.964	148.657	137.903	130.454	125.321	121.625	118.854	116.713	115.004
1200	160.576	152.316	141.623	134.215	129.111	125.436	122.681	120.552	118.853
1300	163.927	155.710	145.073	137.705	132.628	128.972	126.231	124.114	122.423
1400	167.051	158.875	148.291	140.958	135.906	132.268	129.541	127.434	125.752
1500	169.977	161.839	151.303	144.005	138.976	135.355	132.640	130.543	128.869
1600	172.728	164.626	154.136	146.870	141.863	138.258	135.555	133.467	131.800
1700	175.324	167.255	156.809	149.573	144.587	140.997	138.306	136.226	134.566
1800	177.301	169.258	158.845	151.632	146.662	143.082	140.400	138.327	136.672
1873	177.782	169.745	159.340	152.133	147.166	143.590	140.909	138.838	137.185
1900	180.115	172.108	161.742	154.562	149.614	146.051	143.381	141.317	139.670
2000	148.768	140.355	129.464	121.920	116.721	112.978	110.171	108.003	106.273
	10nm	15 nm	20 nm	30 nm	40 nm	50 nm	100 nm	200 nm	CaO(bulk)
1000	104.864	100.511	98.262	95.973	94.812	94.110	92.696	91.983	91.264
1100	113.613	109.315	107.095	104.835	103.688	102.996	101.599	100.895	100.185
1200	117.470	113.196	110.988	108.741	107.601	106.912	105.523	104.823	104.117

1300	121.047	116.796	114.600	112.364	111.230	110.545	109.163	108.467	107.765
1400	124.382	120.152	117.967	115.743	114.614	113.932	112.557	111.864	111.165
1500	127.506	123.295	121.120	118.906	117.783	117.104	115.736	115.046	114.350
1600	130.443	126.251	124.085	121.881	120.762	120.087	118.724	118.038	117.345
1700	133.215	129.040	126.883	124.688	123.574	122.901	121.545	120.861	120.171
1800	135.325	131.163	129.013	126.825	125.715	125.044	123.692	123.010	122.323
1873	135.839	131.680	129.532	127.346	126.236	125.566	124.214	123.533	122.846
1900	138.329	134.186	132.046	129.868	128.762	128.095	126.748	126.070	125.385
2000	104.864	100.511	98.262	95.973	94.812	94.110	92.696	91.983	91.264

Table S3 The Gibbs free energy (G) for (CaO)4, CaO(bulk) and nano-CaO

T	(1/4)$(CaO)_4$	2 nm	3 nm	4 nm	5 nm	6 nm	7 nm	8 nm	9 nm
1000	-1976218.206	-1976258.794	-1976311.340	-1976347.739	-1976372.819	-1976390.879	-1976404.418	-1976414.877	-1976423.228
1100	-1976233.300	-1976273.050	-1976324.510	-1976360.157	-1976384.719	-1976402.406	-1976415.665	-1976425.908	-1976434.086
1200	-1976248.804	-1976287.720	-1976338.102	-1976373.001	-1976397.048	-1976414.365	-1976427.346	-1976437.375	-1976445.381
1300	-1976264.683	-1976302.771	-1976352.080	-1976386.237	-1976409.772	-1976426.721	-1976439.425	-1976449.240	-1976457.077
1400	-1976280.910	-1976318.174	-1976366.417	-1976399.835	-1976422.861	-1976439.443	-1976451.873	-1976461.476	-1976469.142
1500	-1976297.461	-1976333.905	-1976381.087	-1976413.770	-1976436.290	-1976452.507	-1976464.663	-1976474.055	-1976481.553
1600	-1976314.314	-1976349.943	-1976396.068	-1976428.020	-1976450.036	-1976465.890	-1976477.774	-1976486.956	-1976494.286
1700	-1976331.450	-1976366.267	-1976411.342	-1976442.565	-1976464.079	-1976479.572	-1976491.185	-1976500.157	-1976507.321
1800	-1976348.855	-1976382.863	-1976426.891	-1976457.389	-1976478.403	-1976493.536	-1976504.880	-1976513.644	-1976520.641
1873	-1976362.960	-1976396.324	-1976439.517	-1976469.438	-1976490.054	-1976504.900	-1976516.029	-1976524.626	-1976531.491
1900	-1976366.511	-1976399.714	-1976442.699	-1976472.475	-1976492.992	-1976507.767	-1976518.842	-1976527.398	-1976534.229
2000	-1976384.406	-1976416.807	-1976458.754	-1976487.811	-1976507.832	-1976522.249	-1976533.057	-1976541.407	-1976548.073

	10nm	15 nm	20 nm	30 nm	40 nm	50 nm	100 nm	200 nm	CaO(bulk)

1000	-1976430.025	-1976451.026	-1976461.874	-1976472.916	-1976478.520	-1976481.905	-1976488.729	-1976492.170	-1976495.637
1100	-1976440.743	-1976461.311	-1976471.934	-1976482.748	-1976488.236	-1976491.551	-1976498.235	-1976501.604	-1976505.000
1200	-1976451.898	-1976472.035	-1976482.436	-1976493.023	-1976498.396	-1976501.641	-1976508.185	-1976511.483	-1976514.809
1300	-1976463.455	-1976483.163	-1976493.342	-1976503.704	-1976508.963	-1976512.139	-1976518.544	-1976521.772	-1976525.026
1400	-1976475.383	-1976494.665	-1976504.624	-1976514.762	-1976519.907	-1976523.014	-1976529.280	-1976532.439	-1976535.623
1500	-1976487.656	-1976506.514	-1976516.254	-1976526.169	-1976531.201	-1976534.240	-1976540.368	-1976543.457	-1976546.571
1600	-1976500.253	-1976518.688	-1976528.210	-1976537.903	-1976542.822	-1976545.793	-1976551.784	-1976554.804	-1976557.848
1700	-1976513.151	-1976531.167	-1976540.472	-1976549.944	-1976554.751	-1976557.654	-1976563.509	-1976566.460	-1976569.435
1800	-1976526.336	-1976543.933	-1976553.022	-1976562.274	-1976566.969	-1976569.805	-1976575.524	-1976578.406	-1976581.312
1873	-1976537.078	-1976554.341	-1976563.258	-1976572.335	-1976576.941	-1976579.724	-1976585.334	-1976588.162	-1976591.012
1900	-1976539.790	-1976556.970	-1976565.844	-1976574.877	-1976579.461	-1976582.230	-1976587.813	-1976590.627	-1976593.464
2000	-1976553.499	-1976570.264	-1976578.924	-1976587.738	-1976592.212	-1976594.914	-1976600.362	-1976603.108	-1976605.877
